# Cognitive rehabilitation in bipolar spectrum disorder: A systematic review

**DOI:** 10.1016/j.ibneur.2024.04.001

**Published:** 2024-04-10

**Authors:** Mahdiye Sarrafe Razavi, Mazyar Fathi, Elham Vahednia, Amir Rezaei Ardani, Sara Honari, Farzad Akbarzadeh, Ali Talaei

**Affiliations:** Psychiatry and Behavioral Sciences Research Center, Mashhad University of Medical Sciences, Mashhad, Iran

**Keywords:** Bipolar disorder, Cognitive rehabilitation, Neurocognitive deficits

## Abstract

**Background and objectives:**

Neurocognitive deficits in bipolar disorder (BD) have a negative impact on the quality of life, even during the euthymic phase. And many studies conducted to improve cognitive deficits in bipolar disorder. This systematic review aims to summarize studies on cognitive rehabilitation (CR) conducted in bipolar patients and evaluate its impact on neurocognitive deficits. The primary objective is to explore how CR interventions can enhance cognitive functioning, treatment outcomes, and overall quality of life in this population.

**Methods:**

A comprehensive search was conducted on PubMed, Google Scholar, Scopus, Embase, and PsycINFO databases from 1950 to 2023, following the 2015 PRISMA-P guidelines, using search terms related to BD and CR.

**Results:**

The initial search yielded 371 titles across the five databases. After applying inclusion and exclusion criteria through screening, a total of 23 articles were included in the study. The selected articles evaluated verbal memory, attention, executive functions, and social cognition.

**Conclusion:**

The findings suggest that CR can be an effective treatment approach for bipolar patients, aimed at enhancing their cognitive abilities, treatment outcomes, and overall quality of life. The primary finding of this study indicates that cognitive-behavioral therapy (CBT) protocols, skill training, and homework exercises, which offer a daily structure, social support, and opportunities for exchanging coping strategies, are more effective in enhancing cognitive functions. However, it is important to acknowledge the notable limitations of this review. Firstly, we did not assess the methodological rigor of the included studies. Additionally, there was a lack of detailed analysis regarding specific cognitive rehabilitation approaches that adhere to core CR principles, resulting in increased heterogeneity within the reviewed studies.

## Introduction

Bipolar disorder (BD) is a serious mental condition that has been identified as the 17th leading cause of disability among all diseases (Carvalho et al., 2020). Cognitive dysfunction affects approximately 70% of BD patients, even in the euthymic phase (Bora et al., 2016, Ehrminger et al., 2021, Tsapekos et al., 2022), significantly impacting daily functioning. Neurocognitive domains most affected by BD include verbal memory, attention, executive functions (such as behavior inhibition and cognitive flexibility), and social cognition (e.g., theory of mind and emotion recognition) (Kahani et al., 2013, Gomes et al., 2017, Ershad Sarabi et al., 2020, Liu et al., 2021). The impairments observed could be attributed to neurodevelopmental factors like genetic vulnerability, inflammatory and metabolic factors, brain structural and functional variables as well as the progression of the illness, including the detrimental impact of recurring mood episodes and the long-term use of medication (Tsapekos et al., 2019). Furthermore, numerous studies have established a strong relationship between neurocognitive deficits in BD and impairment in daily living activities, social interactions, and occupational functioning (A Martinez-Aran et al., 2007, Martínez-Arán et al., 2011, Bonnin et al., 2013, Akbarzadeh et al., 2023). Although pharmacological treatments improve clinical symptoms in BD, their effectiveness has been limited due to side effects (Talaei et al., 2016, Xu et al., 2020). For instance, sodium valproate has been reported to worsen cognitive conditions in some cases (Ebrahimi et al., 2014). Moreover, the use of multiple types of medication and the prescription of multiple antipsychotics are on the rise, resulting in notable drug interactions and increased occurrence of side effects (Singh et al., 2023) Given the impact of neurocognitive deficits on BD outcomes, cognitive rehabilitation (CR) strategies (MacPherson et al., 2021) have emerged as potential adjunctive treatments. In recent years, numerous studies have concentrated on adjunctive treatment in bipolar disorder (BD). One such systematic review conducted by Bella et al. examined the effects of cognitive remediation and functional remediation, revealing positive outcomes across various cognitive domains. Particularly, improvements were noted in working memory, problem-solving, and processing speed. (Bellani et al., 2019). A different systematic review conducted by Tsapekos et al. explored the potential of cognitive enhancement interventions. The studies included in this review suggest that such interventions may have positive effects on cognitive and functional outcomes, although the evidence remains inconclusive. The relationship between cognitive improvement and functional outcomes remains unclear, and there is a lack of research exploring the mechanisms underlying this transfer.in general CR programs primarily focus on cognitive improvement and daily life functioning, encompassing psychoeducation, coping strategies, and more. The literature on the application of CR in psychiatry mainly focuses on schizophrenia and related disorders (Wykes and Spaulding, 2011, Li et al., 2014). In addition, there are different CR methods, that may have distinct effects on cognition function, and previous studies didn’t compare types of cognitive enhancers, or intervention frequency. So there exists a gap in knowledge regarding the efficacy and methodology of CR specifically in BD. This includes aspects such as intervention frequency, context, total duration, types of cognitive enhancers (computer-based and/or paper-and-pencil), and the approach (functional adaptation and/or general stimulation and/or specific cognitive training, and other skill training). Therefore, this review aims to provide a comprehensive analysis of the existing data regarding the application of cognitive remediation (CR) in bipolar disorder (BD).

## Methods

### Search strategy

This systematic review study followed the PRISMA-P 2015 statement guidelines (Moher et al., 2015, Talebi et al., 2022). For conducting and reporting the review process, the search was performed using the following keywords: "Cognitive rehabilitation OR cognitive remediation OR Cognitive treatment OR Cognitive therapy OR Cognitive training OR cognitive rehabilitation" AND "bipolar disorder." The search was conducted in databases including PubMed, Google Scholar, Scopus, Embase, and PsycINFO, covering articles published from 1950 to 2023.

### Data sources, selections of studies, and data extraction

There were no restrictions on publication years or countries. Two independent researchers screened and selected studies based on title, abstract, and full text. Additionally, the reference lists of the identified articles were reviewed to ensure all relevant studies were included. The inclusion was: (a) The review included studies that involved participants diagnosed with bipolar disorder according to the criteria outlined in DSM-III, DSM-III-R, DSM-IV, or DSM-V, (b) The studies included in this review involved a normal control group without any psychiatric or cognitive problems or reporting cognitive rehabilitation pre-and post-intervention. (c) articles published in English, (d) original studies that specifically focused on bipolar disorder, and. The exclusion criteria were: (a) case reports, editorial letters, and methodological studies, and (b) studies involving fewer than seven subjects.

The selected studies were evaluated based on participant characteristics, intervention features, assessment measures, and outcomes. Participant characteristics included the type of bipolar disorder, sample size, sex, and age (Table 1). Intervention features such as type of intervention, duration of each treatment session, interval between treatment sessions, total intervention time, and intervention design (individual, group, or mixed) were extracted (Table 2). The interventions were categorized as specific or non-specific training, specific training aims to enhance cognitive functions, while non-specific training focuses on improving general skills. Specific training targeted cognitive functions such as organization, attention, and planning, using paper and pencil, and computer-based tools. Non-specific training aimed to improve general skills such as stress management, cognitive behavioral therapy (CBT), and functional adaptation in daily life. Assessment measures were categorized into three groups: clinical assessment tools, cognitive assessment tools, and quality-of-life questionnaires. (Table 1). The outcomes of the interventions and any follow-up assessments were summarized in Table 2.

**Table 1:** primary characteristics and assessments of the studies.

study	Participants characteristics	assessment measures			
		Clinical	Cognitive	Quality of life	follow up
Deckersbach et al. (2010)	N=18 (Average age 36.8), BD I, II	YMRS, HAMD	FrSBe, RBANS, ( IM, DM, A, L, VF, D-KEFS (TMT, CS), WTAR	HPQ, LIFE-RIFT	3 months
Lahera et al. 2012	N=37 (Average age 38.73), (BD I,II, 4 SAD), Control: N=32 (Average age 44.29)	YMRS, HAMD	FEIT and FEDT, ER40	AIHQ, HT	-
Meusel et al. (2013)	N= 87 (18 – 60 years), BD or MDD, Control: N=33	YMRS, HAMD	NART, HVLT, BDS, fMRI	-	-
Torrent et al. (2013)	N=239 (18–55 years), BD I, II	YMRS, HAMD	WAIS-III (V, PSI, DSC, SR) WCST, S, F-A-S, C,), TMT, ROCF, CVLT, WMS-III, CPT	FAST	1 year by Bonnin et al. 2016
Preiss et al. (2013)	N=15 (Average age 42.87) depressive phase of BD, Control: N=16 (Average age 45.38)	BI	CFQ (M,A,MF), DEX (ATi, I, C, PP) EMQ, QBP	SQUALA, SOS10,	-
Demant et al. (2013, 2015)	N=40 (18–50 years), BD	PANS, BDI	fMRI, MGHC, PFQ, EPQ, CI(L, W), CFQ, WHOQ, BI, RAVLT, TMT part A & B, WAIS-III, LNS, V, SFT, CFERT, CANTAN(SRT, RVIP, DMS, SWM, EQ, SCAN, RBANS, FERT, SCPFS, CFQ, WSAS, fMRI	FAST, WHOQOL-BrefPSS, EQL	Week 26
Sole et al. (2015)	N=53 (Average age 40), BP II from torrent study	PANS, BDI	fMRI, MGHC, PFQ, EPQ, CI(L, W), CFQ, WHOQ, BI, RAVLT, TMT part A & B, WAIS-III, LNS, V, SFT, CFERT, CANTAN(SRT, RVIP, DMS, SWM, EQ, SCAN, RBANS, FERT, SCPFS, CFQ, WSAS, fMRI	FAST	-
Venza et al. (2016)	N=27 (21–70 years), BD I, II	HAMD	fMRI, MMSE, TOSL, WAIS Similarities	QoL.BD	-
Zyto et al. (2016)	N=12 (18–65 years), BD I	QIDS-SR, ASRM, QBP	WAIS-IV, TMT part A & B & S, RAVLT, RBMT, WCST, BADS, COWAT, D-A-T, CFQ	CFQ, ASRM, QIDS-SR, DART, FAST	3 months
Bonnin et al. (2016)	N=188 (18–55 years), Data from torrent study with a high level of neurocognitive deficits	QIDS-SR, ASRM, QBP	WAIS-IV, TMT part A & B & S, RAVLT, RBMT, WCST, BADS, COWAT, D-A-T, CFQ	FAST	-
Bonnin et al. (2016)	N=239 (18–55 years), BD I, II	QIDS-SR, ASRM, QBP	WAIS-IV, TMT part A & B & S, RAVLT, RBMT, WCST, BADS, COWAT, D-A-T, CFQ	FAST	1-year follow-up of data of torrent study
Veeh et al. (2017)	N=26 (18–60 years), euthymic	PANAS, MADRS	Mini-ICF-App, TOL, FLEI, TAP, MWT-B, CVLT, S	WHOQOL-BREF	3 months
Lewandowski et al. (2017)	N=72 (18–50 years), BD	PANS, YMRS,HAMD MADRS	MATRICS	-	-
Gomes et al. (2017)	N= applicable (18–55 years), BD I, II	BIS-11, HIS	BRIAN, KADI, MR, WASI,CANTAB, MOT, RVP, RTI, SSP, SWM, OTS, PRM, DMS, AST, ERT	WHOQOL-bref, FAST	-
Gomes et al. (2019)	N=39, BD I, II		MOT, RVP, RTI, SSP, SWM, OTS, PRM, DMS, AST, ERT	QOL	12 weeks
Yang LL et al. (2019)	N=52, BD	YMRS, HDRS	MCCB	-	-
Bernabei, et al. (2020)	N=54 (18–50 years), Euthymic BD I, II	YMRS, HDRS, LSP, BIS-11	RCFT-C & DR, VS, TMT part A & B, SDT, RAVLT-DR, WCST, PVF	-	-
Ott et al. (2021)	N=61, BD	YMRS, HDRS	WAIS-III, RVP, SWM, DS		6 months
Strawbridge et al. (2021)	N=60 (18–65 years), Euthymic	HAMD, YMRS	DSST, FAS, PDQ, SS, DS, VPA1, VPA2, WASI, IQ	EQ-5D-3 L	12 weeks
Tsapekos et al. (2022)	N=80 (18–65 years), Euthymic	HAMD, YMRS,TOPF, FAST, GAS	DSC, DS, VPA2, Hotel test	-	-
Lubbers et al. (2022)	N=49 (age ≥ 18), BD I, II	IDS-C, YMRS, RRS, RPA-NL	BFT, VAS	-	15 months
van den Berg et al. (2023)	N=62, BD I, II	ASRM, QIDS-SR, BAI, ALS-18, Life-Rift, BHS	VAS, MICQ-BD	-	1. 8 weeks 2. 16 weeks

Abbreviations: YMRS: Young Mania Rating Scale, HAMD: Hamilton Depression Rating Scale, FrSBe: Frontal Systems Behavior Rating Scale, RBANS: Repeatable Battery of the Assessment of Neuropsychological Status, IM: Immediate Memory, DM: Delayed Memory, A: Attention, L: Language, VF: Visuospatial Functioning, D-KEFS: Delis-Kaplan Executive Functioning System, TMT: Trail Making Test, CS: Card Sorting, WTAR: Wechsler Test of Adult Reading, HPQ; Healthy Performance Questionnaire, LIFE-RIFT: The Longitudinal Interval Follow-up Evaluation - Range of Impaired Functioning Tool, FEIT and FEDT: Face Emotion Identification Task and the Face Emotion Discrimination Task, ER40: Emotion Recognition-40 task, AIHQ: Ambiguous Intentions Hostility Questionnaire, HT: Hinting Task, NART: National Adult Reading Test, HVLT: Hopkins Verbal Learning Test, BDS: Brock, Dechert and Scheinkman test, fMRI:, Functional magnetic resonance imaging, WAIS-III: Wechsler Adult Intelligence Scale Third Edition, V: Vocabulary, PSI: Processing Speed Index, DSC: Digit-Symbol Coding, SR: Symbol Search, WCST: Wisconsin Card Sorting Test, S: Stroop Color-Word Interference Test, F-A-S: Verbal phonemic test, C: Categorical (animal naming), ROCF: Rey-Osterrieth Complex Figure, CVLT: California Verbal Learning Test, WMS-III: Wechsler Memory Scale Third Edition, CPT: Continuous Performance Test, CFQ: Cognitive Failures Questionnaire, M: Memory, MF: Motor Function, DEX: perception The Dysexecutive Questionnaire, ATi: Abstract thinking, I: Impulsivity, C: Confabulation, PP: Planning Problems, EMQ: Everyday Memory Questionnaire, QBP: Questionnaire for Bipolar Disorder, SQUALA: Subjective Quality of Life Analysis, SOS-10: Schwartz Outcome Scale, PANS: Positive and Negative Affect Scales, BDI: Beck depression inventory, MGHC: Massachusetts General Hospital Cognitive, PFQ: Physical Functioning Questionnaire, EPQ: Eysenck Personality Questionnaire, CI (L, W): Coping Inventory for Stressful Situations (Language, and Words), WHOQ: World Health Organization Quality of life, RAVLT: Rey Auditory Verbal Learning Test, LNS: letter Number Sequencing, SFT: semantic fluency task, CFERT: Computerized Facial Expression Recognition Task, CANTAB: Cambridge Neuropsychological Test Automated Battery, SRT: Situation Reaction Test, RVIP: Rapid Visual Information Processing, DMS: Delayed Matching to Sample, SWM, Spatial Working Memory, EQ: Emotional Intelligence test, SCAN: Schedule for Clinical Assessment in Neuropsychiatry, RBANS: Repeatable Battery for the Assessment of Neuropsychological Status, FERT: Facial Expression Recognition Test, SCPFS: Self-reported Cognitive and Psychosocial Function, WSAS: Work and Social Adjustment Scale, WHOQOL-bref: World Health Organization Quality of Life Brief Version, EQL:, European Quality of Life 5 Dimensions 3 Levels, RCFT: Rey Complex Figure Test, NES: Neurological Evaluation Scale, MMSE: Mini Mental State Exam, TOSL: Test of Scientific Literacy Skills, QoL.BD: Quality of Life in Bipolar Disorder, QIDS-SR: Quick Inventory of Depressive Symptomatology–Self-Report, ASRM: Altman Self-Rating Mania Scale, RBMT: Rivermead Behavioral Memory Test, BADS: Behavioural Assessment of Dysexecutive Syndrome test, COWAT: Controlled Oral Word Association Test, DAT: semantic fluency for patients with Dementia of the Alzheimer's Type, DART; Dutch Adult Reading Test, PANAS: Positive and Negative Affect Schedule scale, MADRS: Montgomery Asberg Depression Rating Scale, Mini-ICF-APP: Social Functioning Scale, TOL: Tower of London Test, FLEI: complaints of cognitive disturbances questionnaire, TAP: Test of Attentional Performance, MWT-B: German Multiple-choice vocabulary intelligence test, MATRICS: Measurement and Treatment Research to Improve Cognition in Schizophrenia, ERP: Event Related Potential, BIS 11: Barratt Impulsiveness Scale 11, BRIAN: Biological Rhythms Interview of Assessment in Neuropsychiatry, KADI: Knowledge about Affective Disorders Interview, WASI: Wechsler Abbreviated Scale of Intelligence, MOT: Motor screening Task, RVP: Rapid Visual Information Processing, RTI: Reaction Time, SSP: Spatial Span, OTS: One Touch Stockings of Cambridge, PRM: Pattern Recognition Memory, DMS: Delayed Matching to Sample, AST: Attention Switching Task, ERT: Emotion Recognition Task, HDRS: Hamilton Depression Rating Scale, MCCB: MATRICS Consensus Cognitive Battery, VS: Visual Search, SDT: Symbol Digit Test, PVF: Phonological Verbal Fluency, LSP: Life Skills Profile, DS: Digit Span, DSST: Digit Symbol Substitution Test, FAS: verbal fluency test, PDQ: perceived deficits questionnaire, SS: Symbol Search, VPA: Verbal Paired Associates assessment, IQ: Intelligence Quotient test, TOPF: Test of Premorbid Functioning, FAST: Functioning Assessment Short Test; GAS: Goal Attainment Scale, IDS-C: Inventory of depressive symptomatology—clinician administered, RRS: Ruminative Response Scale, RRS-EXT: Ruminative Response Scale—Extended Version, RPA-NL: Responses to Positive Affect—dutch version, BFT: Breathing focus task, VAS: Visual Analogue Scales, BAI: Beck Anxiety Inventory, ALS-18: Affect Lability Score Short Version, BHS: Beck Hopelessness Scale, MICQ-BD: Mental Imagery in Bipolar Disorder Questionnaire.

**Table 2:** Results and interventions of the studies.

Study	Intervention characteristic			Outcomes
	Type of interventions		Duration of each session, the interval, the total duration, and the design	
	Specific	non-specific intervention		
Deckersbach et al. (2010)	organization, planning, time management, memory, attention (paper-pen)	CBT, CS, mood, monitoring, residual depressive symptoms	14 individual treatment sessions of 50-minutes for 4 months	− Reduction of residual depressive symptoms − Improving executive performance and the quality of life − Increasing occupational and psychosocial performance
Lahera et al. (2012)	SCIT (computer-based), emotional perception, attributional style, and theory of mind abilities	-	24 weekly 60-minute treatment sessions in groups of 12 cases	− Improving emotional perception and theory of mind − Reducing hostile attribution
Meusel et al. (2013)	PSSCogRehab (computer-based), processing speed, attention, verbal memory, executive function, working memory	-	-1-hour treatment sessions, three times a week for 10 weeks -fMRI	− Improving the backward digit span − Increasing patients' activation in lateral and medial prefrontal, superior temporal, and lateral parietal regions − Increasing recollection-related activation in the bilateral hippocampus
Torrent et al. (2013)	Organization, memory, attention, problem-solving, reasoning, multitasking (paper-pen)	Strengthen performance in daily routine	21 weekly 90-minute treatment sessions	− Increasing occupational and psychosocial performance − No reduction in subclinical depressive symptomatology − No improvement in the cognitive domain
Preiss et al. (2013)	CogniFit (computer-based)	Adherence monitoring by checking participants' progress through phone calls every 2 weeks	20–30 minute treatment sessions, three times a week for 8 weeks	− Reducing the level of depression − Improvements in switching, divided attention, and the global executive control score − No improvements in mood − Reducing cognitive deficits, dysexecutive incidents, and difficulty in daily routine performances
Demant et al. 2013(protocol), 2015 (results)	RehaCom, memory, executive function, social cognition, working memory, emotional face processing, attention, (computer-based)	Mindfulness exercises, CS, psychoeducation	12 treatment sessions of 120 minutes including 15 minutes of rest in groups of 5–8 cases	Improvement of mental acuity, verbal fluency and psychological quality of life (despite the overall negative result of the trial)
Sole et al. (2015)	Subanalysis in patients with BPII from the data of torrent et al.	Repeat	Repeat	− Increasing functional outcome − Reducing sub-depressive symptoms
Venza et al. (2016)	Attention, integrative reasoning, executive function, memory, SMART (computer-based)	-	-2-hour treatment sessions, once a week for 4 weeks in groups of 5–7 cases -fMRI	− enhanced cognition (improved executive functions, complex abstraction) − Increased resting CBF in the right prefrontal cortex
Zyto et al. (2016)	memory, working memory, attention, speed of information processing, planning	Combined group and individual functional, compensatory techniques, psychoeducation	12 treatment sessions (6 in group and 6 in individual sessions), each session with two 45 -minute sections with 15 minutes of rest between each session	Improving psychosocial performance (autonomy) and and occupational function
Bonnin et al. (2016)	Subanalysis in patients with cognitive impairment from the data of torrent et al.	Repeat	Repeat	− Improving psychosocial performance and verbal memory − Improving delayed free recall in CVLT
Bonnin et al. (2016)	1-year follow-up of Torrent et al. data	Repeat	Repeat	Improving psychosocial performance (autonomy)
Veeh et al. (2017)	HAPPYneuron (computer-based)	Motivating the patients with positive feedback	12 weekly 90-minute treatment sessions in groups of 4–6 cases	− Reducing symptoms of depression − Improving working memory, problem-solving, and divided attention − No changes in psychosocial performance and quality of life − Lack of correlation between subjective cognitive complaints and objective test performance
Lewandowski et al. (2017)	PositScience (computer-based)	-	1-hour treatment sessions, three times a week for 24 weeks	Cognitive improvement
Gomes et al. (2017)	memory, attention, executive functioning (not applicable)	CBR: CR+CBT	12 weekly 90-minute treatment sessions in groups of 8–10 cases	not published
Gomes et al. (2019)	memory, attention, executive functioning (not applicable)	Group-based CR	12 weekly group treatment sessions	− Improving reaction time, visual memory, facial expression recognition − No changes in functional and quality of life scores
Bernabei, et al. (2020)	CRIIT(COGPACK software), Video Game (computer-based)	motivational meetings, psychoeducation and rehabilitation interventions	20 individual 1 hour sessions, 3 times per week	− Significant Improvement in domains related to executive functions, attention, memory, functioning and impulsivity − ……
Ott et al. (2021)	ABCR		10 weeks (2 × weekly) ABCR sessions	− No effects of ABCR on cognitive composite score − Significant effects on executive performance and subjective cognitive performance
Strawbridge et al. (2021)	CIRCuiTs (computer-based)	Face-to-face, phone call, in person sessions and individual practice	A12-week treatment sessions of 20–40 hours	Improving in IQ, working memory, executive function, psychosocial performance, and goal achievement in CIRCUiTS participants
Tsapekos et al. (2022)	CIRCuiTs (computer-based)	one-on-one hourly sessions, in person or remotely sessions, supplementary independent practice sessions at home	2–3 hours' treatment sessions per week for 40 weeks	− Correlation of reduction in functional problems with the time spent in each session in contact with the therapist − Correlation of improvement in goal achievement with the number of helpful strategies used per session − Significant effects of CR on all outcomes influenced by number of sessions
Lubbers et al. (2022)	MBCT	Pharmacotherapy, Psychoeducation and Self-management	8 weekly 2.5 hours' treatment sessions, one 6 hours silent day, 45 minutes daily home practice	− Reducing depressive rumination and negative intrusive thoughts
van den Berg et al. (2023)	ImCT, Psychoeducation	-	Twelve 1-hour treatment sessions of ImCT or six 2-hour group sessions of psychoeducation for 4 weeks	− Reducing mood instability, levels of mania, depression, and anxiety in both ImCT and Psychoeducation − Significant reduction in daily measures of depression and anxiety, reduction in hopelessness, and reduction in intrusive and problematic imagery in ImCT compared to Psychoeducation
				-

Abbreviations: CBT: Cognitive Behavioral Therapy, SCIT: Social cognition and interaction, RCFT: Rey Complex Figure Test, SMART: Strategic Memory Advanced Reasoning Training, CBF: cerebral blood flow, CVLT: California Verbal Learning Test, CBR: Cognitive brain reserve, CR: Cognitive Rehabilitation, CRIIT: Cognitive Remediation in Integrated Treatment, ABCR: Action-Based Cognitive Remediation, MBCT: Mindfulness-Based Cognitive Therapy, ImCT: Imagery-focused Cognitive Therapy.

## Results

**Fig. 1:**
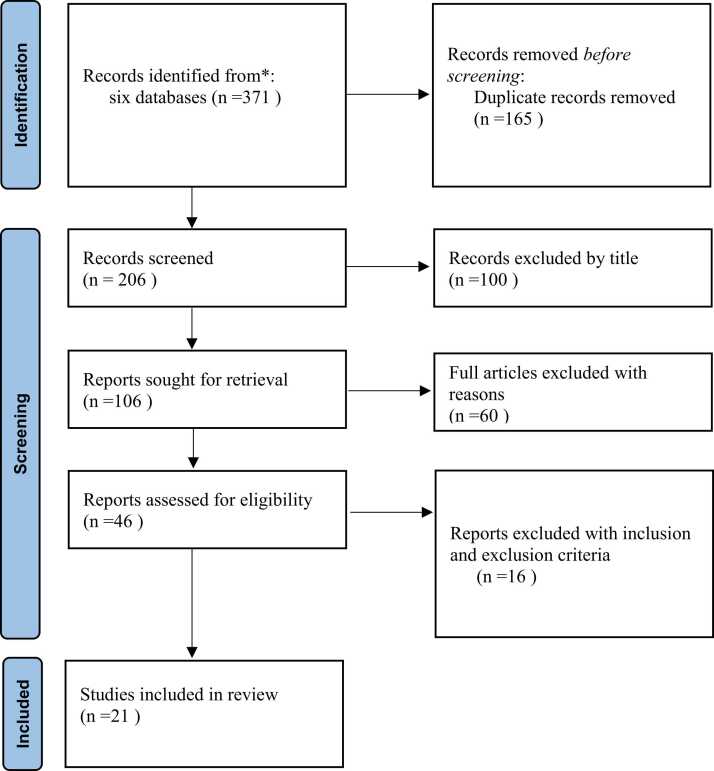
Flow diagram of the methodology and selection process used in this study, in base of the prisma guidelines.

In the initial search, 371 titles were found. After checking for duplications, 206 articles were selected. The abstracts of these 206 articles were reviewed, and 23 articles were screened for their relevance to cognitive rehabilitation (CR) and bipolar disorder (BD) (Fig. 1). The main aim of this review was to evaluate the efficacy of a CR program in BD management, and the overall results demonstrated positive treatment effects of CR in BD. However, the results varied based on participants' characteristics, clinical measures, quality of life measures, and cognitive measures (Table 1). The type of cognitive rehabilitation methods was also evaluated, including specific and non-specific intervention, the duration of each treatment session, the interval of treatment sessions, the total duration of the intervention, and the design of the intervention (individual, group, or mixed) (Table 2). These factors contribute to a better understanding of the effectiveness of CR in BD.

Out of the five review articles published between 2015 and 2022, three reported a lack of CR benefit in BD. However, review articles that focused exclusively on CR showed promising effects in BD.). Among the 5 review articles, only one focused specifically on CR in BD. Kluwe-Schiavon et al. (2013) reviewed four studies on CR in BD (A Martinez-Aran et al., 2007, Lahera et al., 2013, Preiss et al., 2013, Torrent et al., 2013) and reported that CR interventions could improve executive performance in BD (Kluwe-Schiavon et al., 2013). The other review articles discussed various treatment modalities related to the effect of CR in BD. anchez-Moreno et al. (2010) focused on treatment approaches for improving functional impairments in BD and concluded that CR programs should be integrated into the treatment protocol for patients (Sanchez-Moreno et al., 2010). Miziou et al. (2015) reviewed different psychosocial interventions in BD and reported no significant effects of CR. (Miziou et al. 2015). MacQueen et al. (2017) reviewed various treatment strategies recommended for alleviating cognitive impairments in mood disorders and concluded that more well-designed controlled studies are needed to demonstrate the efficacy of CR in mood disorders (MacQueen and Memedovich, 2017). Miskowiak et al. (2022) provided effective treatments and methodological recommendations by examining cognitive modification, and pharmacological interventions, (Miskowiak et al. 2022). They reviewed four studies that reported the effect of CR as the most consistent cognitive benefit and as an adjunct to pharmacotherapy in partially improved BD patients. (Lewandowski et al., 2017, Gomes et al., 2019, Ott et al., 2021, Strawbridge et al., 2021). Regarding clinical outcomes and quality of life, most studies showed the effectiveness of cognitive training such as paper and pencil exercises, and computer-based programs in improving cognitive function in BD. For example, Strawbridge and colleagues conducted a randomized trial and demonstrated the positive effect of their protocol, which included individual therapy, cognitive therapy, and computer-based therapy, on cognitive and psychosocial performance in BD cases (Strawbridge et al., 2021). Other studies by Meusel et al. and Venza et al. showed that computer-based cognitive training for 10 weeks (3 times per week) and 4 weeks (2 hours per week), respectively, improved cognitive deficits in BD patients (Meusel et al., 2013, Venza et al., 2016). The Cognitive Remediation in Integrated Treatment (CRIIT) protocol was used as a specific add-on cognitive rehabilitation intervention for euthymic bipolar disorder patients in the Bernabei et al. study. The protocol consisted of 20 individual 60-minute sessions, conducted three times per week, using the COGPACK software. The results of the program showed significant improvements in executive functions, attention, memory, functioning, and impulsivity (Bernabei et al., 2020). Additionally, fMRI studies indicated increased prefrontal and parietal functions, as well as cerebral blood flow (CBF) in specific brain regions during cognitive performance in BD (Lewandowski et al., 2017). Lubbers et al. demonstrated that mindfulness-based cognitive therapy (MBCT) significantly reduced rumination and negative intrusive thoughts in BD patients (Lubbers et al., 2022). Furthermore, van den Berg et al. reported a significant reduction in depressive symptoms, anxiety, hopelessness, and intrusive and problematic imagery after a four-week intervention using imagery-based cognitive therapy (van den Berg et al., 2023).

In some studies, CR not only improved cognitive deficits and quality of life but also reduced other clinical symptoms. For example, Deckersbach et al., Preiss et al., and Veeh et al. followed CR protocols for 12 weeks (50 minutes per session), 8 weeks (3 times per week, 20–30 minutes), and 12 weeks (90 minutes per session), respectively, and reported positive outcomes in clinical and quality of life measures (Deckersbach et al., 2010, Preiss et al., 2013, Veeh et al., 2017). Different techniques and approaches were used in these interventions, including cognitive-behavioral therapy (CBT), lifestyle modifications, coping strategies, psychosocial skills, and role-playing (Veeh et al., 2017), and Deckersbach et al. used CBT in their protocols (Deckersbach et al., 2010)(Table 2). Also, in Preiss, et al.'s study, the training of the participants was designed based on the results of the neurocognitive evaluation, and the program was continuously adapted to the strengths and weaknesses of each person (Preiss et al., 2013). Finally, the progress of the participants was monitored, which indicated more motivational goals for the patient and better results in clinical, quality of life, and neurological outcomes. Based, it seems that the design and content of the therapy session are most important. Furthermore, some contextual factors (personal and environmental), including dysfunctional attitudes, motivation, resilience, self-efficacy, or poor perception of social support may affect functional outcomes.

Several studies highlighted the importance of inclusion and exclusion criteria, as well as the context of the study. Factors such as the age of onset, symptom severity, course of the disease, and social and emotional background can affect the cognitive function of individuals with mood disorders (MacQueen and Memedovich, 2017). For instance, Torrent et al. evaluated CR in BD management using three arms: functional remediation, psychological education, and standard pharmacological therapy (TAU). Their study included 239 euthymic bipolar patients and demonstrated that functional remediation was more effective in improving functioning compared to psychological education. (Torrent, Bonnin et al. 2013). The CR group program included stress management, problem-solving, and communication skills. Functional improvement was more significant using functional remediation compared to psychological training, Psychoeducation is aimed at preventing relapses and only affects the quality of life despite the performance in the short term. Bonnin et al. and Torrent et al. reported that CR effectively improved verbal memory and psychosocial performance in bipolar patients with neurocognitive impairment (Bonnin et al., 2016). Since most of these patients had chronic conditions and multiple episodes, it has been suggested that functional remediation may be effective in the later stages of the disease. Deckersbach et al. found that patients with neurocognitive impairment were less likely to benefit from CR (Deckersbach et al., 2010). A one-year follow-up of Torrent. et al. data by Bonnin. et al. showed a better functional outcome in the functional remediation group in comparison with psychoeducation or TAU groups (Bonnin et al., 2016). Also, a sub-analysis of Torrent. et al. data by Sole et al. showed reduced depressive symptoms and better performance in CR training (Table 2) only in BPII samples (Sole et al., 2014). Gomez et al. reported improvements in reaction time, visual memory, and emotion recognition as a result of CR intervention. By designing 12 group-based CR sessions, they evaluated the effectiveness of CR on cognitive and functional impairment in BD (Gomes et al., 2019). Demant et al. (2015) designed a 12-week treatment program consisting of 120-minute sessions. They utilized various skills and techniques, including coping strategies, mindfulness, homework, and others (Table 2). However, their results did not show any significant effects except for an improvement in subjective sharpness and mental acuity based on a self-assessment tool (Demant et al., 2015). Yang et al. (2019) evaluated the effects of 10 consecutive days of repetitive transcranial magnetic stimulation (rTMS) on clinical manifestations and cognitive functions in BD patients. The results demonstrated an improvement in cognitive function following the intervention (Yang et al., 2019). Also, the studies of Bersani et al. (2017) and Minichino et al. (2015) conducted studies that indicated simultaneous prefrontal-excitatory and cerebellar-inhibitory transcranial direct current stimulation (tDCS) in euthymic BD patients may lead to better neurocognitive performance (Minichino et al., 2015, Bersani et al., 2017). In a study conducted by the current authors (2020), tDCS was shown to improve mood and cognitive deficits, specifically in writing abilities, in bipolar patients, Combining a group and individual format in CR may increase treatment efficiency through a tailor-made approach. This approach allows for a group process while also addressing individual goals. A group setting creates a sense of connection among group members, increasing motivation and participation (Yang et al., 2019). According to Lahera et al. (2013), social cognition and interaction training improved emotional perception and decreased depressive symptomatology (Lahera et al., 2013).

## Discussion

The present systematic review aimed to summarize the available evidence on cognitive rehabilitation (CR) in bipolar disorder (BD) and its impact on neurocognitive deficits. The review included a total of 23 articles that evaluated various cognitive domains, such as verbal memory, attention, executive functions, and social cognition in bipolar patients by CR interventions.

The results of this review indicate that CR can be an effective treatment approach in bipolar patients, with the potential to enhance their cognitive abilities, treatment outcomes, and overall quality of life. Several studies included in this review reported positive effects of CR interventions on cognitive functioning, daily functioning, and quality of life in bipolar patients. These findings are consistent with previous research suggesting that neurocognitive deficits in BD can be improved through targeted cognitive interventions (Kluwe-Schiavon et al., 2013, Tamura et al., 2021). In summary, CR has the potential to improve the quality of life and functional outcomes in BD. Factors such as the completion of sessions, session duration, and therapist presence can enhance the effectiveness of CR. Different types of interventions, including cognitive training, psychoeducation, and psychosocial skills training, have shown promising results in improving cognitive deficits and clinical symptoms in BD. However, the effectiveness of CR may vary depending on factors such as participant characteristics, the specific cognitive domains targeted, and the design and context of the intervention. Further research is needed to better understand the optimal parameters and mechanisms underlying the efficacy of CR in BD (Tsapekos et al., 2022). The cognitive domains most commonly targeted in the included studies were verbal memory, attention, executive functions, and social cognition which is line with other studies (King et al., 2019, Chen et al., 2021, Gillissie et al., 2022). These domains are known to be significantly impaired in bipolar patients and have a considerable impact on their daily functioning and overall well-being (Bora et al., 2016, Ehrminger et al., 2021, Tsapekos et al., 2022). The use of various intervention strategies, such as paper-and-pencil training, computer-based programs, transcranial direct current stimulation (tDCS), and repetitive transcranial magnetic stimulation (rTMS), highlights the diversity of approaches in CR interventions for bipolar patients. It is worth noting that the reviewed studies used different methodologies and outcome measures, making it challenging to directly compare their results. Additionally, the duration, frequency, and intensity of the CR interventions varied across studies. Therefore, it is difficult to determine the optimal parameters for CR in bipolar patients. Future research should aim to establish standardized protocols for CR interventions in BD, including the duration and frequency of sessions, the specific cognitive domains to target, and the most effective intervention modalities. The current review also identified several gaps in the existing literature. The majority of the included studies focused on bipolar patients with BD type I or II, leaving a gap in knowledge regarding the effectiveness of CR interventions in other subtypes of BD. Additionally, there is limited research on the long-term effects of CR interventions and their sustainability over time. Future studies should address these gaps by including diverse samples of bipolar patients and conducting follow-up assessments to evaluate the durability of the cognitive improvements achieved through CR.

Furthermore, the review highlighted the need for more well-designed controlled studies to provide robust evidence for the efficacy of CR in BD, especially the evaluation of long-term CR effects in BD. Although the majority of the reviewed studies reported positive effects of CR interventions, some studies did not find significant cognitive benefits. This inconsistency may be attributed to methodological limitations, small sample sizes, or other confounding factors. Therefore, larger randomized controlled trials with rigorous study designs are necessary to ascertain the true effectiveness of CR in bipolar patients.

## Conclusions

In conclusion, this systematic review suggests that CR can be a valuable adjunctive treatment approach for bipolar patients, aiming to improve their cognitive functioning, treatment outcomes, and overall quality of life. Probably, a three-month design (about 90 minutes of weekly training sessions) of cognitive training including CBT protocols, skill trainings, and homework exercises provides a daily structure, social support, and the possibility of exchanging coping strategies. More effective CR protocols could be reached by mindfulness and combined individual and group therapy sessions. In addition, the result of this review study indicated that a longer duration of treatment sessions does not lead to a greater cognitive improvement (Meusel et al., 2013, Preiss et al., 2013, Demant et al., 2015). However, further research is needed to establish standardized protocols, optimize intervention parameters, and ensure the long-term sustainability of cognitive improvements. Also, this review has notable limitations. We did not evaluate the methodological rigor of included studies, which is essential for assessing the validity and reliability of findings. Our synthesis also lacked granular analysis of specific cognitive rehabilitation approaches adhering to core CR principles, resulting in increased heterogeneity. Finally, the absence of meta-analytic techniques limits conclusions about the overall strength of effects. Future work should systematically appraise methodological quality, analyze outcomes by intervention type, and apply meta-analytic synthesis to provide more rigorous evidence on the efficacy of cognitive rehabilitation for bipolar disorder.

## CRediT authorship contribution statement

**Ali Talaei:** Investigation, Methodology, Supervision. **Farzad Akbarzadeh:** Methodology, Software. **Mazyar Fathi:** Data curation, Investigation, Methodology, Supervision, Writing – original draft, Writing – review & editing. **Amir Rezaei Ardani:** Data curation, Investigation. **Sara Honari:** Writing – review & editing. **Mahdiye Sarrafe Razavi:** Conceptualization, Investigation, Methodology, Visualization, Writing – original draft. **Elham Vahednia Vahednia:** Investigation, Methodology, Software.

## Declaration of Competing Interest

The authors declare that they have no financial or personal interests that could influence the work reported in this paper.

## Data Availability

The data that support the findings of this study are available on request from the corresponding author. The data are not publicly available due to [restrictions e.g. their containing information that could compromise the privacy of research participants].
